# Structural analysis of functional connectivity related to memory reveals network changes in memory function over the life span

**DOI:** 10.1186/1471-2202-12-S1-P71

**Published:** 2011-07-18

**Authors:** Franziska Matthäus, Jan-Philip Schmidt, Traute Demirakca, Carsten Diener

**Affiliations:** 1 Center for Modeling and Simulation in the Biosciences (BIOMS), Heidelberg University, Heidelberg, Germany; 2 Institute for Applied Mathematics, Heidelberg University, Heidelberg, Germany; 3 Central Institute of Mental Health, Mannheim, Germany

## 

We address the question how the function of memory changes over the life span. For this we analyse fMRI data collected for healthy individuals ageing 20 up to over 80 years during two different memory tasks. Working memory is assessed through a 2-back task, episodic memory through active remembering of autobiographic events. From the fMRI data we construct networks of functional connectivity by correlating the fMRI signal of every pair of voxels. For the resulting networks we compute structural parameters and identify important anatomic structures. The analysis reveals that the density and size of the networks increases with age, related to a stronger and wider distributed brain activity during memory tasks in elderly subjects, and a more specific and localized activity in younger individuals. We also observe a shift in the localization of regions that are involved in memory processing, and also in the connectivity patterns. While all individuals exhibit a strong connection between the frontal and the occipital lobes, elderly subjects are additionally characterized by a strong connection to parietal areas. Our analysis shows that additional brain areas become involved in memory function as age increases, and that also connectivity patterns change, hinting at either compensatory mechanisms or in artifacts related to erroneous co-activation.

